# Impact of Heat and Mass Transfer during the Transport of Nitrogen in Coal Porous Media on Coal Mine Fires

**DOI:** 10.1155/2014/293142

**Published:** 2014-06-25

**Authors:** Bobo Shi, Fubao Zhou

**Affiliations:** ^1^School of Safety Engineering, China University of Mining and Technology (CUMT), Xuzhou 221116, China; ^2^Key Laboratory of Gas and Fire Control for Coal Mines, CUMT, Xuzhou 221116, China; ^3^National Engineering Research Center for Coal and Gas Control, CUMT, Xuzhou 221116, China

## Abstract

The application of liquid nitrogen injection is an important technique in the field of coal mine fire prevention. However, the mechanism of heat and mass transfer of cryogenic nitrogen in the goaf porous medium has not been well accessed. Hence, the implementation of fire prevention engineering of liquid nitrogen roughly relied on an empirical view. According to the research gap in this respect, an experimental study on the heat and mass transfer of liquid nitrogen in coal porous media was proposed. Overall, the main mechanism of liquid nitrogen fire prevention technology in the coal mine is the creation of an inert and cryogenic atmosphere. Cryogenic nitrogen gas vapor cloud, heavier than the air, would cause the phenomenon of “gravity settling” in porous media firstly. The cryogen could be applicable to diverse types of fires, both in the openings and in the enclosures. Implementation of liquid nitrogen open-injection technique in Yangchangwan colliery achieved the goals of fire prevention and air-cooling. Meanwhile, this study can also provide an essential reference for the research on heat and mass transfer in porous media in the field of thermal physics and engineering.

## 1. Introduction

Liquid nitrogen, a safe, highly efficient, clean, easily obtained, and low-temperature refrigerant, has been now widely used in biology, medical treatment, animal husbandry, food, metallurgy, electronics, aerospace and cryogenic industry, and other fields. Liquid nitrogen has the dual role of cooling and inerting; (i) heat absorption of vaporization can make the fuel combustible temperature drop below ignition temperature, and (ii) cryogenic nitrogen after vaporizing expansion could make the oxygen content in the atmosphere significantly reduced. Therefore, liquid nitrogen is a highly efficient fire extinguishing agent. Previous studies showed that liquid nitrogen can rapidly and effectively put out the sodium fire [1], for which water and carbon dioxide extinguishing agents fail to achieve the effect. Liquid nitrogen can also be employed to extinguish isopropanol [2], ethanol, propanol and diesel [3], and other oil pool fires, as well as building fires [4], thus avoiding damage to property caused by water extinguishing agent.

However, unlike building or oil pool fire, subsurface fire features a fire source concealed which is difficult to approach or timely detection of fire sources (such as fire areas of goaf). Therefore, it is very difficult to extinguish the fire; causing underground fires may continue for months or even years. Currently, coal mine fire continues to be one of the major hazards during the global mine production [5–7], which directly threatens mine safety production and causes severe hazards including serious air pollution, water pollution, ecological disturbance, and geological disasters, in addition to the potential massive loss of energy resources. Fire-proof techniques as grouting, foam, or colloid injection were not in a position to function ideally, due to the limited coverage [8, 9], stopping the extinguishment substance from reaching the fire location. The grouting material tends to inflow into the working face, which causes the pollution of the working section of mine. Use of water or chemical fire extinguishers may cause unwanted side effects, such as extensive “water damage,” air pollution, or underground water contamination. With the rapid development of coal mine exploitation, a fully submerged fire extinguishing technique that can meet the needs of coal mine fire extinguishing in large-scale exploitation is needed. Gaseous nitrogen has been used to prevent and extinguish fires as an efficient inert gas. This technique is of advantages of quenching the fire zones, preventing gases from the explosion, and is of wide-range diffusion. However, the technique has low specific heat and inefficient heat transfer; therefore, the normal cooling effect makes it necessary to supplement it with some other fire extinguishing measures to control the fire. This extends the fire extinguishing period, leading to failure to meet the anticipated fire extinguishing period in the field. In a word, current extinction agents and methods are neither always effective nor always environmentally safe.

Confronted with such a difficult problem, fortunately, the application of liquid nitrogen injection was confirmed to be an effective technique in the field of coal mine fire prevention and achieved quite good results in the prevention of subsurface waste bank coal spontaneous combustion [8–10]. However, the mechanism of heat and mass transfer of cryogenic nitrogen in the complex environment of goaf porous medium has not been well accessed. Hence, the implementation of fire prevention technology and engineering of liquid nitrogen roughly relied on an empirical view. According to the research gap in this respect, an experimental study on the heat and mass transfer of liquid nitrogen in loose coal body was proposed, in order to reveal the fire prevention law of cryogenic nitrogen and provide guidance for implementation of liquid nitrogen fire prevention and extinguishing technology and engineering in in situ testing. Meanwhile, this study can also provide an important reference for the research on heat and mass transfer in porous media in the field of thermal physics and engineering.

## 2. Experimental Setup

**Figure 1:**
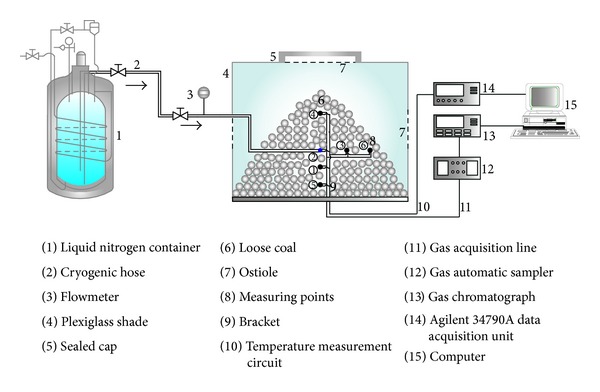
Experimental setup of heat and mass transfer of liquid nitrogen in porous media.

The experimental platform (as shown in Figure 1) was primarily composed of a self-pressurized liquid nitrogen container, a cryogenic hose, a loose coal body system, a temperature acquisition system, an oxygen concentration acquisition system, and a computer. Among them, the effective volume of self-pressurized liquid nitrogen container was 100 L, the standard working pressure was 0.1 MPa, and the daily evaporation rate was less than 1.3%. Measuring ranges of the flow meter were from 0 to 5.0 L/min with accuracy of ±0.1 L/min. Medium temperature inside the flow meter ranged from −200°C to +80°C. Diameter of liquid nitrogen cryogenic hose was 25 mm. Medium temperature inside the cryogenic hose could range from −196°C to +200°C.

Loose coal body system included a low-temperature-resistant plexiglass shade, loose coal, and pedestal. The shade was a cube of side length of 1000 mm, the round hole which is just above it had a diameter of 300 mm, and the four round openings around the wall were of 20 mm. Heat and mass transfer regularity of liquid nitrogen in porous media was derived depending on the simulation of the low-temperature-resistant plexiglass shade opening. When simulating open perfusion liquid nitrogen, all openings of the cover body above and around were opened. On the contrary, all the openings were closed in the condition of closed perfusion liquid nitrogen. The loose body medium was selected for coal, a kind of microporous medium material, which was also a kind of accumulation porous media from the macrolevel. Industrial analysis results of coal sample were shown in Table 1.

Selecting an average coal particle size of 5 mm to 10 mm, 10 mm to 15 mm, and 15 mm to 20 mm in a sieve, three groups of coal particles piled into the height of 400 mm coal loose. The coal loose was positioned in the plexiglass shade. Voidage parameter which is marked as *χ* was employed to characterize the three groups of accumulation body loose, as follows:

(1) $$\begin{array}{c} \chi =\frac{V_{n}-\left(1+k\right)V_{0}}{V_{n}}, \end{array}$$

where *V*_*n*_ is the apparent volume of the pile. *V*_0_ is the volume of a single coal particle. *K* is the space factor, whose value is 0.4 in this experiment. The cryogenic hose perpendicular to this plane was placed in the coal loose body horizontally. Hose outlet was connected to screens for the purpose of liquid nitrogen releasing uniformity in the lateral. A two-dimensional coordinate system was set up to describe all measuring points with the nitrogen injection port as the coordinate origin. Coordinates of all measuring points in order were 1# (0, −80), 2# (0, 0), 3# (80, 0), 4# (0, 160), 5# (0, −160), and 6# (160, 0). The experiment was carried out at room temperature of 10°C, and oxygen concentration was 20.95% in the atmosphere. Liquid nitrogen flow was controlled at 1.0 L/min approximately. The experimental scheme was shown in Table 2. Based on the experimental method of two-dimensional transient simulating, heat and mass transfer regularity of liquid nitrogen in coal body loose porous medium was revealed in this paper.

Determination of temperature and oxygen concentration was the primary data collection of the simulation experiments. For real-time measurement of temperature, temperature acquisition system consisted of a copper constantan thermocouple and Agilent 34790A data collector determination. In addition, the temperature of thermocouple was calibrated and it ranged from 77 K to 300 K before the formal experiment. In this experiment, the diffusion process of nitrogen was inverted by the measured amount of the decrease in oxygen concentration. Oxygen gas samples were gathered through the inner diameter of 3 mm fine brass. Meanwhile, the end of fine brass and thermocouple tip were bound together and placed in the loose coal body, in order that both the measured temperature and oxygen concentration were from the same point at the same time. The other end of slender brass was connected to the sample automatic inhalation device of gas chromatograph. Eventually, oxygen concentration data analysis was obtained by the GC4000A gas chromatograph. Before the start of the experiment, calibration and verification of temperature and gas concentration in porous media were made in the condition of room temperature and atmospheric pressure, ensuring the repeatability of the experimental system and the accuracy of experimental data.

## 3. Results

### 3.1. Instantaneous Temperature of Each Experiment

**Figure 2:**
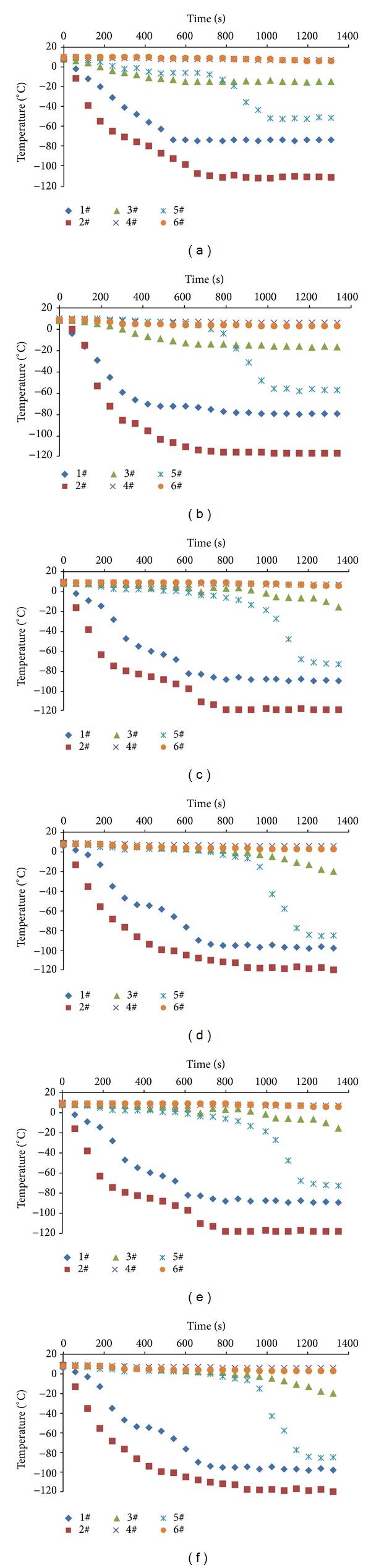
Temperature variation of measuring points dependent on the time.

As can be observed in Figure 2, the temperature change tendencies of the same measuring points were essentially the same among the six sets of experiments. Temperature of 1# and 2# measuring points reduced most significantly, while that of 3# measuring point showed a smaller change during the nitrogen injection. Meanwhile, 5# measured temperature decreased suddenly in the later nitrogen injection period. 4# and 6# measured temperature changed slightly in the whole process of nitrogen injection. In other words, cryogenic nitrogen flowed downward with limited horizontal dispersion. From the experimental results of the average coal particle size of 17.5 mm in A group and B group, temperature of 3# measuring point located at the side of the nitrogen injection outlet dropped to 0°C in 200 s and to −20°C in the later stage of the experiment. Comparatively speaking, in the light of the experimental results of the average coal particle size of 7.5 mm in E group and F group, temperature of 3# measuring point decreased to below 0°C until the time of 1000 s. That said, voidage of porous media hit the rate of the liquid nitrogen heat transfer. Through the experimental comparison of open and closed nitrogen injection effect, the trend of measuring points temperature varied substantially the same. Hence, the results showed that the two kinds of conditions had little effect on the heat transfer of liquid nitrogen in porous loose media.

### 3.2. Instantaneous Variation of Oxygen Concentration of Each Experiment

**Figure 3:**
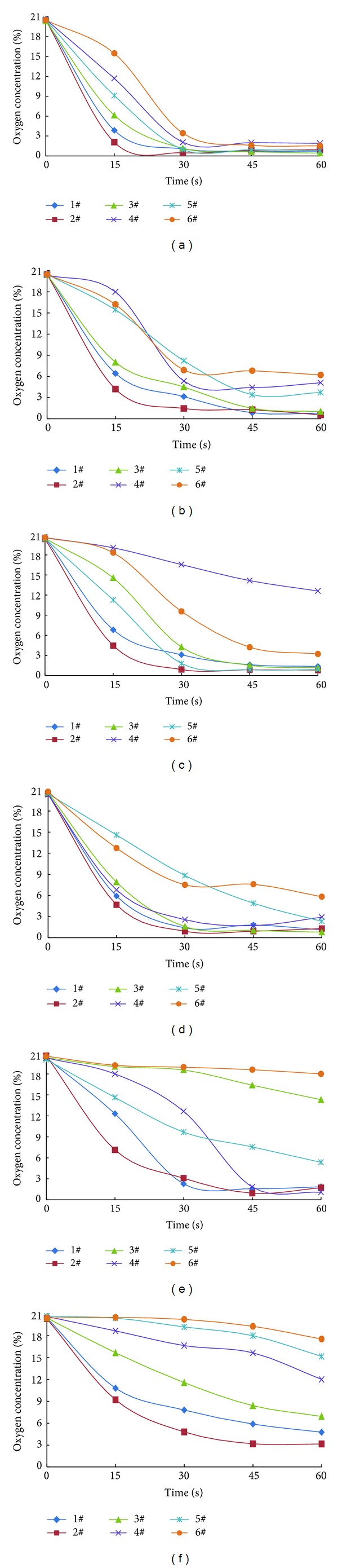
Oxygen concentration variation of measuring points dependent on the time.

It is clearly observed from Figure 3 that nitrogen quickly filled the entire plexiglass shade space in a short time because of the rapid vaporization and expansion of liquid nitrogen. Therefore, the oxygen concentration of all measuring points decreased significantly in a short period. Under the condition of liquid nitrogen perfusion in limited space, vaporized nitrogen played a good role in forcing warm oxygen gas out. The oxygen content decreasing sharply in the container indicated that liquid nitrogen infusion could effectively inhibit the coal spontaneous combustion, so as to avoid the mine fire. From the experimental results of the average coal particle size of 7.5 mm in E group and F group, the oxygen concentration reduced slightly, which might be related to the small coal particle or porous medium voidage.

It is a known fact that heat and mass transfer in porous media is an extremely complex physical problem. In particular, liquid nitrogen transporting in porous media would cause complicated energy and mass transfer processes and heat and mass transfer processes, including liquid nitrogen phase change, heat conduction, convection, gas seepage, gas diffusion, nitrogen adsorption or desorption on coal, and other complex processes. Hence, macroscopic analysis results of the experiment were obtained just from the perspective of fire prevention in this paper in order to give the implementation basis for fire prevention technology and engineering. Consolidated results of Figures 2 and 3 could be drawn that the main mechanism of liquid nitrogen fire prevention technology in the coal mine was the creation of an inert and cryogenic atmosphere.

## 4. Discussion

### 4.1. Voidage

**Figure 4:**
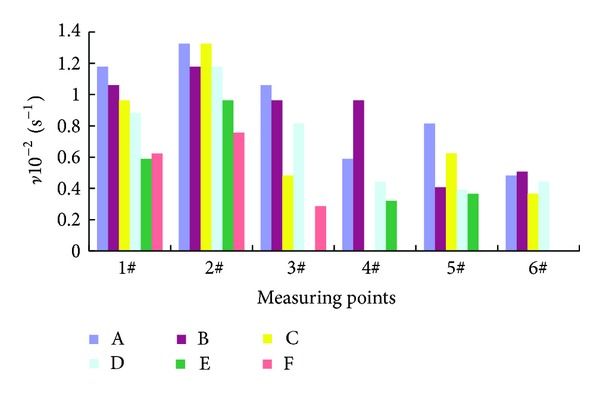
The change rate of the nitrogen concentration *ν*.

From the above analysis, liquid nitrogen fire prevention technology dominated in inerting effect aspect. Referring to the inert gas inerting indicators for fire prevention in coal mines, oxygen concentration used for inert or explosion inhibition should be less than 12% in the atmosphere. According to the experimental conditions, the definition of oxygen concentration reached 10% as the critical concentration of liquid nitrogen fire prevention technology. The parameter *ν* in (2) was defined as liquid nitrogen vapor transport rate in loose porous coal under the experimental conditions. The experimental results were shown in Figure 4:

(2) $$\begin{array}{c} \nu =100\frac{\Delta C}{\Delta t}, \end{array}$$

where *ν* is the liquid nitrogen vapor transport rate in loose porous coal in s^−1^. Δ*C* is the difference between the initial oxygen concentration and the critical concentration in %. And Δ*t* is the time that reached the critical inerting concentration in s.

For different particle sizes of loose coal, the migration rate of temperature and nitrogen concentration field was also different, as shown in Figure 4. Blank data in Figure 4 showed that the nitrogen concentration of measuring points did not reach the inert index. The smaller the equivalent diameter of the coal particle is, the worse the air permeability would become, thus delaying the transmission rate of nitrogen vapor penetration and temperature steam. Taking the 1# measuring point, for example, for A group, C group, and E group experiment with open liquid nitrogen injection, comparison of parameter *ν* read as follows: *ν*_A_ > *ν*_C_ > *ν*_E_, while, for the B group, D group, and F group experiment with closed liquid nitrogen injection, comparison of parameter *ν* was as follows: *ν*_B_ > *ν*_D_ > *ν*_F_.

### 4.2. Fire District Environment

The experiment simulated both the open and closed liquid nitrogen injection in two different ways, comparing to the temperature and oxygen concentration variation under two kinds of conditions of different nitrogen injection in loose coal body. As can be seen from Figure 4, open and closed liquid nitrogen injection in the two approaches had less impact on the performance of liquid nitrogen vapor diffusion in loose coal under our experimental conditions. In other words, it provided a basis for the coal mine fire prevention engineering field of open liquid nitrogen perfusion.

### 4.3. Vertical and Horizontal Dispersion

Cryogenic nitrogen alone, injected at a temperature of −196°C in dry coal refuse, acted like a liquid, flowing downward with limited horizontal dispersion [11, 12]. A comparative analysis of 1# and 3# measuring points in Figure 4 indicated that the diffusion rate of nitrogen vapor vertically down was 1.1 to 2.2 times that of the horizontal direction. The comparative analysis of 5# and 6# measuring points indicated that the diffusion rate of nitrogen vapor vertically down was 1.7 times that of the horizontal direction.

However, if the refuse was wet, ice formed during cryogenic injection could contain and direct the flow of the liquid nitrogen. That might be the reason why the oxygen concentration decreased slightly from the experimental results of the average coal particle size of 7.5 mm in E group and F group.

As the liquid nitrogen evaporated, the expanding gas acted like a piston, forcing warm air out of the porous bed. The diffusion of nitrogen vapor after cryogenic liquid vaporization was more complex than normal nitrogen. Cryogenic nitrogen gas vapor cloud, heavier than the air, would cause the phenomenon of “gravity settling” firstly. The density of liquid nitrogen vaporized gas was available calculated in accordance with (3). Due to atmospheric turbulence, air was sucked into the cloud interior, and low-temperature heavy gas cloud would be heated and converted to the upright gas (smaller than the density of the air) proliferation:

(3) $$\begin{array}{c} \rho_{g}=130.8\text{E}-6\cdot \frac{Mp}{T}, \end{array}$$

where *M* represents the molecular weight of nitrogen in 28 g/mol.  *p* is the absolute pressure of the gas in Pa. *T* is the calculated temperature in *K*.

Thus, the density of the gas was inversely proportional to temperature under the condition of constant pressure. When *ρ*_*g*_ > *ρ*_air_, diffusion performance of cryogenic nitrogen vapor was similar to the heavy gas dispersion characteristics. When *ρ*_*g*_ < *ρ*_air_, nitrogen vapor showed nonheavy gas floating characteristics.

## 5. Case Demonstration

Yangchangwan colliery, a large coal mine designed with a capacity of 15 million t/a, is located in the Ningxia Hui Autonomous Region of China. It has a 12# mining area of 1# well which belongs to class II heat harm area, its average geothermal gradient is 3 to 4.5°C /100 m, and its return air temperature of working face is as high as 34°C in summer. Spontaneous combustion period of the coal seam is from 1 to 3 months, a minimum of 23 days, with coal spontaneous combustion grade level I (causing spontaneous combustion easily) and ignition point of 305°C. Under the dual threat of ground temperature harm and coal spontaneous combustion disaster, mine fire prevention problem is very prominent and thorny.

In the 120204 working face of Yangchangwan colliery, with a 5.6 m high-height mining design, fire prevention situation was particularly grim. It can be seen from Table 3 that the scale of spontaneous combustion hazardous area of working face was extraordinary large. In particular, the width of spontaneous combustion hazardous zones near the air inlet side of goaf was more than 145 m, and that of air-leakage zones was 20 m approximately. The width of spontaneous combustion hazardous zones in the middle of goaf was about 100 m, and that of air-leakage zones was 35 m approximately. Compared with general fully mechanized working face, the spontaneous combustion hazardous zones of mine waste bank high-height mining face were relatively large-scale ones. The voidage of the high-height mining goaf was relatively large, increasing the leakage intensity of goaf.

**Figure 5:**
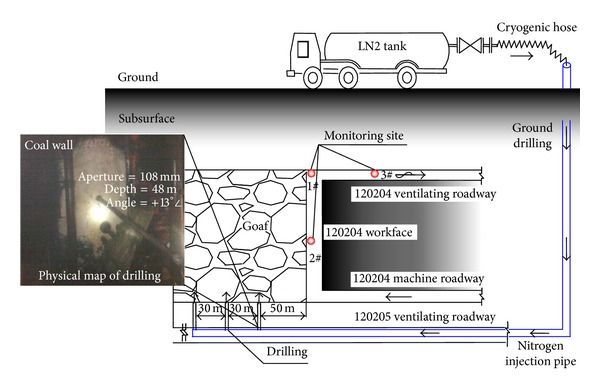
Open fire prevention and control technology with liquid nitrogen.

Therefore, liquid nitrogen fire prevention technology of ground drilling injection was implemented in order to ensure the safety of production during the mining face recovery. Meanwhile, an open liquid nitrogen injection mode was carried out in the working face for the purpose of routine artificial production. The process of liquid nitrogen injection technology by surface drilling was that liquid nitrogen of tank was transported by the hose and ground drilling to the underground chamber. After decompression of the pressure relief valve, liquid nitrogen flowed through the stainless steel seamless pipeline and drilling and entered the waste bank of the working face finally. Liquid nitrogen fire prevention system and technological process were illustrated in Figure 5.

The so-called liquid nitrogen open-injection technique was to inject cryogenic nitrogen into the mined-out areas, so as to achieve the goal of rapid decreasing of goaf temperature and the amount of oxidizing gas in the rear of working face. By adopting this antifire technology, not only the oxygen concentration of mined-out fire hazardous areas was in the security value below 10%, but also the reduced oxygen concentration of return air was less than 1% during the liquid nitrogen injection, so as to prevent the asphyxia of workers in the working face. However, reports on direct applications (dumping) of liquid nitrogen on open fires cannot be readily found in the open scientific literature. This work intends to fill this void.

Three liquid nitrogen injecting boreholes were arranged in the industrial test, respectively, from the air inlet side of goaf of 50 m, 80 m, and 110 m. Layout principle of drilling is referred to in Table 3. According to the aforementioned experimental results, construction parameters for drilling were the angle of +13°, 48 m drilling depth, and 108 mm aperture. In the centre, upper corner and return airway of the working face were, respectively, provided with 1#, 2#, and 3# monitoring points, as shown in Figure 5.

For liquid nitrogen open-injection technique, the maximum allowable nitrogen injection intensity was calculated in accordance with the complete leakage of nitrogen in the working face from the mined-out areas injection:

(4) $$\begin{array}{c} Q_{\max}=60\times Q\times \frac{C_{1}-C_{2}}{C_{2}}. \end{array}$$

In (4), *Q*_max⁡_ is the maximum allowable nitrogen injection intensity of goaf according to the oxygen concentration of 18% in the air return way. *Q* is the air quantity of working face in 1513 m^3^/min. *C*_1_ is the initial oxygen concentration of working face in 21%. *C*_2_ is the allowable oxygen concentration of working face in 18%. Because 1 ton liquid nitrogen vaporization could form about 800 m^3^ gas nitrogen, the maximum liquid nitrogen injection intensity of 120204 working face should not exceed 9 t/h through the above calculation.

**Figure 6:**
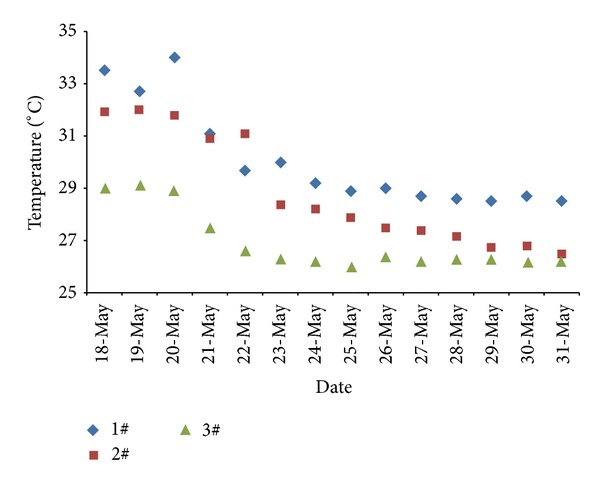
Temperature curve of monitoring sites after liquid nitrogen injection.

From May 20 onwards, the accumulation liquid nitrogen perfusion of 28 t was filled in the 120204 mined-out areas. Analysis of fire suppression effect was through manual sampling, artificial detection method during the liquid nitrogen injection periods. The engineering practice proved that the temperature of the working face dropped from 32°C to 28°C approximately after the implementation of liquid nitrogen open-injection technique. Before and after injection, the temperature curve of liquid nitrogen injection in working face was presented in Figure 6.

**Figure 7:**
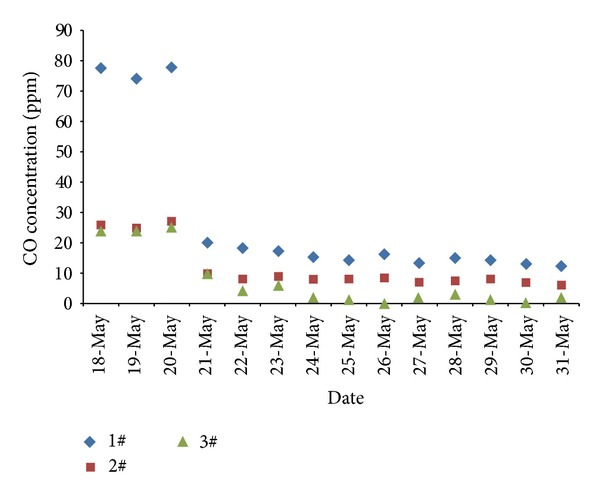
CO concentration of monitoring sites after liquid nitrogen injection.

Before injection measures of liquid nitrogen in the working face, CO concentration of 2# measuring point was approximately 0.0024%, and CO concentration of 1# measuring point was 0.008% approximately, serious threat to the safe production of the coal mine. During the liquid nitrogen injection periods, the CO concentration decreased gradually; 2# measuring point was always less than 0.0008%. CO concentration of 1# measuring point was below 0.0015%, and that of 3# measuring point was always lower than 0.0005%. Liquid nitrogen fire prevention and extinguishing, of significant effect, effectively inhibited the spontaneous combustion process of coal in the coal mine. The CO concentration curve of liquid nitrogen injection in working face was shown in Figure 7. Implementation of liquid nitrogen open-injection technique in Yangchangwan colliery achieved the goals of fire prevention and cooling, also helping to improve the working environment of underground personnel.

## 6. Main Conclusions

1. An experimental study on the heat and mass transfer of liquid nitrogen in loose coal body was proposed, in order to reveal the fire prevention law of cryogenic nitrogen and provide guidance for engineering. Liquid nitrogen transporting in porous media would cause complicated energy and mass transfer processes and heat and mass transfer processes, including liquid nitrogen phase change, heat conduction, convection, gas seepage, gas diffusion, nitrogen adsorption or desorption on coal, and other complex processes. Hence, macroscopic analysis results of the experiment were obtained just from the perspective of fire prevention in this paper in order to provide the implementation basis for fire prevention technology and engineering. The main mechanism of liquid nitrogen fire prevention technology in the coal mine is the creation of an inert and cryogenic atmosphere. The following major fire extinguishing mechanisms act simultaneously: (i) surface cooling because of the very low temperature of the cryogen and the latent and sensible heats; (ii) inerting of the atmosphere, around the fire source, and starving the fire from oxygen; (iii) rapid expansion followed by gravity spread that results in blanketing of the surface with a cloud of gaseous nitrogen, condensed water vapor, and fuel vapors.
2. Cryogenic nitrogen gas vapor cloud, heavier than the air, would cause the phenomenon of “gravity settling” in porous media firstly. The diffusion rate of nitrogen vapor vertically down was 1.7 times that of the horizontal direction approximately under the condition of the experiment. In other words, cryogenic nitrogen flowed downward with limited horizontal dispersion. Hence, when adopting antifire technique of liquid nitrogen, nitrogen injection drilling should locate in a fire zone or slightly above the spontaneous hazardous areas.
3. For different particle sizes of loose coal, the migration rate of temperature and nitrogen concentration field was also different. The smaller the equivalent diameter of the coal particle is, the worse the air permeability would become, thus delaying the transmission rate of nitrogen vapor penetration and temperature steam.
4. In principle, the cryogen may be applicable to diverse types of fires, both in the open areas and in enclosures. Open and closed liquid nitrogen injection in two different ways had less impact on the performance of liquid nitrogen vapor diffusion in loose coal under our experimental conditions. It provided a basis for the coal mine fire prevention engineering field of open liquid nitrogen perfusion. Implementation of open liquid nitrogen injection technique in Yangchangwan colliery achieved the goals of fire prevention and air-cooling and also helped to improve the working environment of underground personnel.

## Figures and Tables

**Table 1:** Industrial analysis of coal sample.

Porosity, %	Proportion, g/cm^3^	Industrial analysis, %			
		Moist	Ash	Volatile matter	Fixed carbon
5.74	1.18	8.71	14.74	31.66	44.89

**Table 2:** Grouping of experimental conditions.

Average particle size *d*, mm	Voidage *χ*, %	Infusion environment	Number
17.5 (15~20)	42.5	Open	(A)
		Closed	(B)
12.5 (10~15)	39.1	Open	(C)
		Closed	(D)
7.5 (5~10)	37.3	Open	(E)
		Closed	(F)

**Table 3:** Spontaneous combustion hazard zone of goaf.

	Air-leakage	Spontaneous combustion	Suffocation
Air inlet side of goaf	0~20 m	20 m~165 m	>165 m
Middle of goaf	0~35 m	35 m~135 m	>135 m
Air outlet side of goaf	0~12 m	12 m~145 m	>155 m
